# Recent progress in thiocarbazone metal complexes for cancer therapy via mitochondrial signalling pathway

**DOI:** 10.3389/fchem.2024.1424022

**Published:** 2024-05-30

**Authors:** Yunyun Zheng, Hangyi An, Jinxu Qi, Jiaming Li

**Affiliations:** ^1^ Medical School of Pingdingshan University, Pingdingshan, China; ^2^ Guangxi Key Laboratory of Green Chemical Materials and Safety Technology, College of Petroleum and Chemical Engineering, Beibu Gulf University, Qinzhou, China

**Keywords:** thiocarbazones, metal complexes, mitochondrial, apoptosis, cell cycle

## Abstract

Mitochondria are the energy factories of cells and are important targets for the development of novel tumour treatment strategies owing to their involvement in processes such as apoptosis, oxidative stress, and metabolic programming. Thiosemicarbazone metal complexes target mitochondria and reduce mitochondrial membrane potential. The breakdown of mitochondrial membrane potential is a key event in the early stage of apoptosis, which releases cytochrome C and other pro-apoptotic factors, activates the intracellular apoptotic enzyme cascade, and eventually causes irreversible apoptosis of tumour cells. Thiosemicarbazone metal complexes targeting the mitochondria have recently emerged as potential antitumour agents; therefore, this review describes the structural diversity of thiosemicarbazone metal [Fe(III), Cu(II), Ni(II), Zn(II), Ga(III), Pb(II), Au(III), and Ir(III)] complexes and explores their anti-tumour mechanisms that target mitochondrial pathways.

## 1 Introduction

Mitochondria, the energy centre of biological cells, play a prominent role in tumour biology, being involved in regulating cell energy metabolism and free radical production and clearance (Zakic et al., 2024). They are also deeply involved in cell apoptosis, autophagy, signal transduction, and other key biological activities (Ye et al., 2019; Wang et al., 2020; Tian et al., 2023; Wan et al., 2024). Mitochondria have therefore become attractive targets for anti-tumour therapies. Drugs designed to target mutations in mitochondrial DNA, or specific mitochondrial proteins, interfere with mitochondrial function in tumour cells without affecting normal cells (Chen et al., 2020; Degitz et al., 2022; Marynowicz et al., 2023). Compounds that open the mitochondrial outer membrane permeability conversion pore and release cytochrome C and apoptosis-inducing factors can be used to program the death of tumour cells (Ahmad et al., 2021; Zhao et al., 2022; Zhou et al., 2023). Metal complexes and small-molecule drugs have been used to increase the level of reactive oxygen species (ROS) in tumour cells, causing excessive oxidative stress that induces apoptosis or autophagy (Chen et al., 2017; Aghvami et al., 2018; Cantoni et al., 2022).

Thiosemicarbazone metal complexes, typically formed by combining metal ions such as copper, zinc, and platinum with thiourea-derived ligands, have attracted extensive attention in the field of anti-tumour research owing to their diverse biological activities and strong potential in anti-tumour applications (Matesanz et al., 2021; Bai et al., 2022; Jain et al., 2023). Thiosemicarbazone metal complexes significantly affect the mitochondrial function by interfering with energy metabolism and promoting ROS production, which together promote tumour cell death via mitochondria-mediated apoptosis (Jiang et al., 2023; Li et al., 2023; Montalbano et al., 2023). Some thiosemicarbazone metal complexes can overcome resistance caused by traditional chemotherapy drugs, providing a new strategy for the treatment of drug-resistant tumours (Gu et al., 2020). Numerous *in vitro* experiments and animal model studies have shown that thiosemicarbazone metal complexes exhibit strong anti-tumour activity against various tumour types, including breast, lung, and liver cancers (Jaragh-Alhadad et al., 2022). The potential of thiosemicarbazone metal complexes as anti-tumour agents that target mitochondria has recently been recognized in pharmacochemistry and oncology research. These complexes induce apoptosis by specifically affecting the mitochondria of tumour cells, resulting in enhanced therapeutic effects and fewer side effects.

## 2 Design principles and structural diversity

**FIGURE 1:**
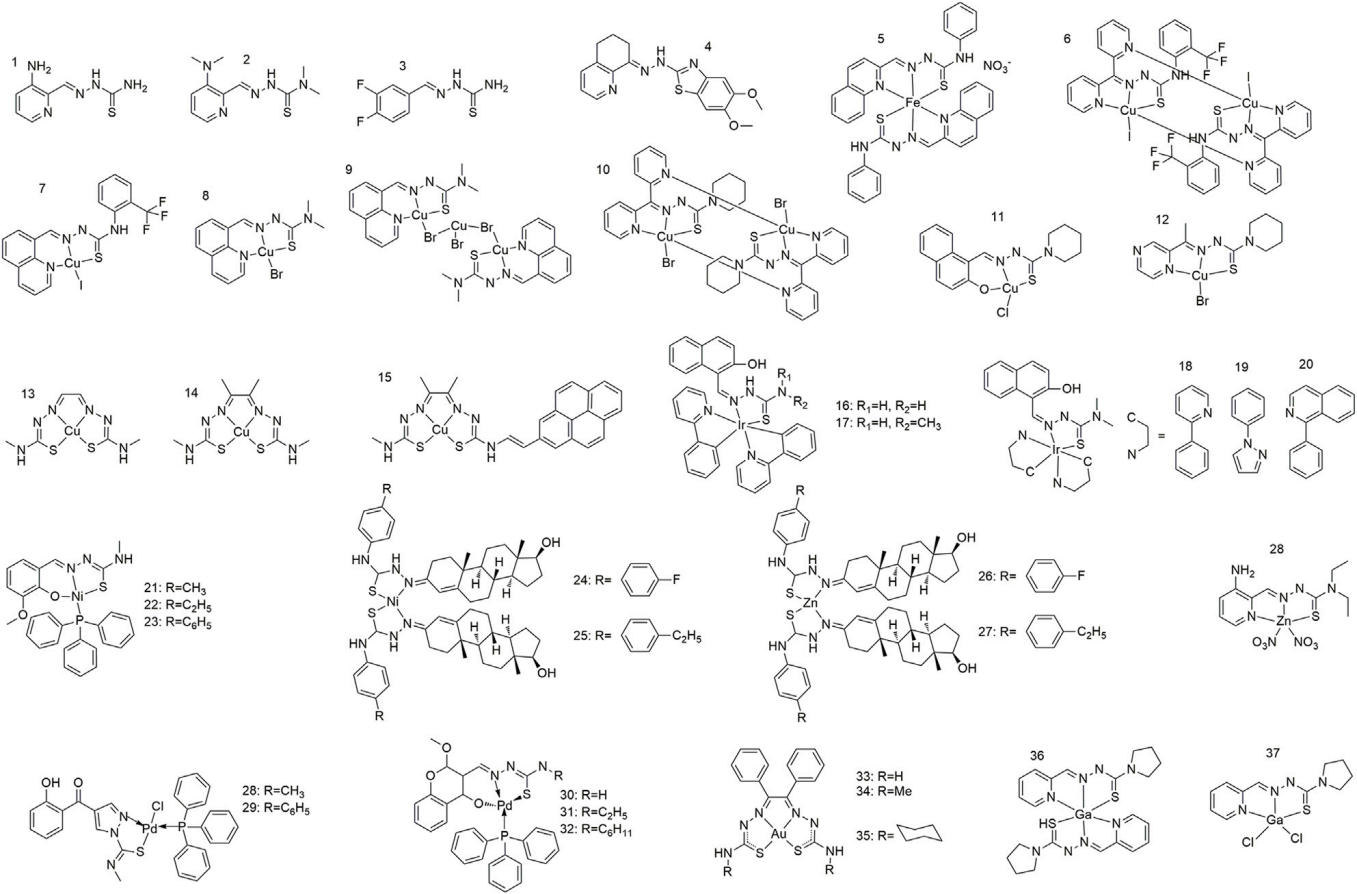
Metal complexes of thiocarbazone for cancer therapy via modulation of mitochondrial signaling pathways.

Thiosemicarbazone ligands (1–4) are typically bidentate or polydentate due to the presence of nitrogen and sulfur atoms in its molecular structure, both of which can serve as coordination sites for forming coordination bonds with metal ions (Zhao et al., 2017; Hager et al., 2018; Chen et al., 2019). Specifically, thiosemicarbazone’s fundamental structure comprises an amino group (-NH_2_) and a thiourea (-NHC(=S)NH_2_) moiety, wherein both the nitrogen and sulfur atoms possess lone pair electrons that can participate in coordination interactions (Jiang et al., 2022; Yang et al., 2023). Ligand adjustability is a critical component in the design of thiosemicarbazone-metal complexes. Changing the substituents on the thiourea groups or introducing different metal ions allows the physical and chemical properties of the complexes to be adjusted, thereby affording complexes with different biological activities (Figure 1). This structural diversity provides a broad scope for optimising the anti-tumour performance. Gou *et al.* synthesised an Fe(III) complex (5) with an α-N-heterocyclic thiocarbazone ligand, which showed relatively strong redox activity, with an Fe(III/II) redox potential similar to that of cellular oxidants and reducing agents (Gou et al., 2016). Zhang *et al.* synthesised two α-N-heterocyclic thiosemicarbazone Cu(II) complexes (6 and 7), which showed superlative *in vitro* inhibitory activity against HCC (human lung adenocarcinoma) cell lines with IC_50_ values of 0.2 and 2 μM, respectively (Zhang et al., 2022). Gu *et al.* successfully synthesised two copper complexes (8 and 9) from N-heterocyclic thiosemicarbazone and CuBr_2_ that exhibited remarkable anticancer and anti-metastasis activities (Gu et al., 2019). These copper complexes exhibit fluorescence between 450 and 600 nm (with an excitation wavelength of 405 nm) and are therefore suitable for cellular localisation studies. Qi *et al.* synthesised three Cu(II) complexes (10, 11, and 12); the antitumour activity of these Cu(II) complexes increased by more than 40 times relative to that of the ligands and exhibited significant pro-apoptotic activity at nanomolar concentrations (Qi et al., 2018). Yoshii *et al.* synthesised a series of positron emission tomography imaging agents for hypoxic tumours based on Cu-bis(N^4^-methylthiosemicarbazone) complexes (13 and 14) (Yoshii et al., 2012; Djoko et al., 2014). Price *et al.* synthesised a Cu(II) pyrene complex (15) incorporating diacetyl-bis (N^4^-methylthiosemicarbazone) in the ligand, which enable the confocal fluorescence imaging of compounds in cells through subcellular localisation (Price et al., 2011). Li *et al.* modified the N-3 hydrogen atoms and structure of a cyclometallized Ir(III) dimer with 2-hydroxy-1-naphthalaldehyde thiocarbazone ligands to afford a series of Ir(III) complexes (16–20), one of which (20) exhibited particularly significant fluorescence and cytotoxicity against cancer cells (Li et al., 2024). Kalaivani *et al.* synthesized and identified a series of novel Ni(II) complexes (21–23) (Kalaivani et al., 2014). They further demonstrated the *in vitro* cytotoxicity of these complexes towards human lung adenocarcinoma (A549) cells by effectively binding to CT-DNA through intercalation. Heng *et al.* coupled testosterone with 4-(4-ethylphenyl)-3-thiosemicarbazone and 4-(4-fluorophenyl)-3-thiosemicarbazone to afford Schiff base ligand that were used to synthesize Ni(II) and Zn(II) complexes (24–27) that were selective for the colorectal cancer cell line HCT 116 (Heng et al., 2020). Qi *et al.* synthesised a Zn(II)-thiosemicarbazone complex with a metal to ligand coordination ratio of 1:1 (27) that exhibited significantly stronger fluorescence and antitumour activity than the ligand alone (Qi et al., 2020). The reaction of 3-formyl chromone with 4-methyl or 4-phenylthiosemicarbazone in the presence of acetic acid afforded two ligands that further reacted with [PdCl_2_(PPh_3_)_2_] to yield two square planar Pb(II) complexes (28 and 29) (Haribabu et al., 2020a). The Michael addition pathway yielded three new Pd(II) complexes (30 and 32), 32 of which exhibited activity against MDA-MB-231 (human breast cancer cell line) and AsPC-1 (human pancreatic cancer cell line) cancer cells with IC_50_ values of 0.5 and 0.9 μM, respectively, and had superior anti-tumour activity to cisplatin (Haribabu et al., 2020b). Qi *et al.* synthesised 2-pyridine-formaldehyde thiocarbamide Ga(III) complexes (36 and 37) with 2:1 and 1:1 ligand:Ga(III) ratios. The antiproliferative activity of the Ga(III) complex with a 1:1 metal/ligand ratio (37) was significantly higher than that with a metal:ligand ratio of 1:2 (36) (Qi et al., 2017).

## 3 Anti-tumour mechanisms targeting mitochondrial pathways

**FIGURE 2:**
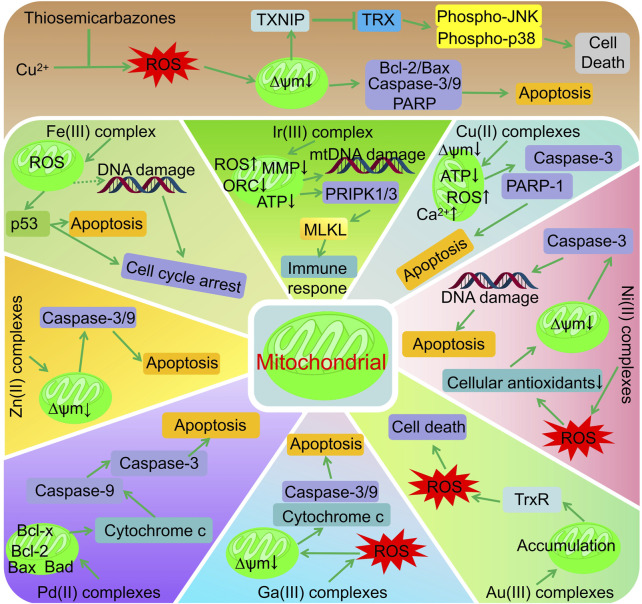
Anti-tumor mechanisms of thiocarbazone metal complexes.

In addition to their role in producing energy, mitochondria also play an important role in regulating apoptosis. The survival and rapid proliferation of tumour cells rely on abnormal mitochondrial functions; therefore, targeting the mitochondria can directly disrupt the energy metabolism of tumour cells and promote tumour apoptosis while minimising the impact on normal cells. Zhao *et al.* demonstrated that the thiosemicarbazone ligands effectively inhibit tumor cell proliferation and invasion by reducing mitochondrial membrane potential, significantly increasing reactive oxygen species content, decreasing the Bcl-2/Bax ratio, and upregulating the expression of p53, Cleaved-Caspase 3, and Cleaved-PARP (Zhao et al., 2017). The antitumour mechanism of thiosemicarbazone metal complexes targeting the mitochondrial pathways is shown in Figure 2. The Fe(II) complex of thiosemicarbazone (5) promotes excessive ROS production in cells, induces p53 phosphorylation, regulates the expression of bcl-2 family proteins, and causes mitochondrial dysfunction. This triggers the release of apoptotic factors such as cytochrome c from the mitochondria, leading to caspase-3/9 cleavage by caspase family proteases, ultimately causing apoptosis (Gou et al., 2016). Zhang *et al.* found that the conformation of DNA can be altered or distorted by copper complexes that bind to DNA mainly in a groove pattern. Two of these complexes cause severe mitochondrial damage by elevating ROS and Ca^2+^ levels, lowering both adenosine triphosphate (ATP) levels and mitochondrial membrane potential (Δψm), and altering mitochondrial morphology (Zhang et al., 2022). Using confocal fluorescence imaging, Gu *et al.* determined that complex 9 primarily accumulates in the mitochondria and promotes HeLa cell apoptosis by reducing the mitochondrial membrane potential and inducing ROS production (Gu et al., 2019). Qi *et al.* observed a similar apoptotic mechanism using complex 10, which causes excessive intracellular ROS, leading to dissipation of the mitochondrial membrane potential and promoting the release of mitochondrial apoptotic factors (Qi et al., 2018). Complex 13 reversibly and competitively binds to ubiquitin-binding sites in mitochondrial Complex I, thereby inhibiting mitochondrial respiration (Djoko et al., 2014). Li *et al.* showed that the Ir(III) complex (20) promoted intracellular ROS production and decreased levels of MMP (mitochondrial membrane potential), OCR (mitochondrial aerobic respiratory oxygen consumption rate), and ATP (adenosine triphosphate) by targeting mitochondria, thereby inducing liver cancer cell necrosis, activating necrosis-related immune responses, and effectively inhibiting tumour growth and metastasis (Li et al., 2024). Kalaivani *et al.* used Ni(II) complexes (21–23) to consume the cellular antioxidant pool (GSH, SOD, CAT, GPx, and GST) via the reaction of ROS and lipid peroxidation, thereby reducing the mitochondrial membrane potential, activating caspase-3, inducing apoptosis in A549 cells, and inhibiting both cell migration and metastasis (Kalaivani et al., 2014). The substitution of testosterone n4 with thiosemicarbazone Ni(II) and Zn(II) complexes (20–23) induces apoptosis via mitochondria-dependent and exogenous apoptotic pathways (Heng et al., 2020). Compared with ligands, Zn(II) complex (27), primarily located in the mitochondria, more effectively reduces the mitochondrial membrane potential, promotes the activation of caspase-3/9, and causes cell cycle arrest and apoptosis (Qi et al., 2020). The anti-tumour mechanism of Pb(II) complexes (28–32) involves the release of cytochrome C, activation of caspase-3, and induction of apoptosis via mitochondria-mediated pathways (Haribabu et al., 2020a; Haribabu et al., 2020b). Rodriguez-Fanjul *et al.* demonstrated that the Au(III) complex (35) was distributed in the cytoplasm and mitochondria of MCF7 cells, thereby inhibiting intracellular TrxR activity, increasing intracellular ROS levels, and inducing cell death (Rodriguez-Fanjul et al., 2018). The Ga(III) complex significantly increased caspase-3/9 activation in NCI-H460 cells via mitochondrial cytochrome C-mediated release (Qi et al., 2017).

## 4 Conclusion and perspectives

The optimisation of the mitochondrial targeting, anti-tumour activity, and biocompatibility of thiosemicarbazone ligands via structural modifications (such as the incorporation of specific functional groups) and the introduction of various metal ions has recently emerged as an area of considerable research interest (Wang et al., 2019; Shyamsivappan et al., 2020; Duan et al., 2022). Some studies have highlighted the high selectivity of these complexes, which selectively recognise and bind to the mitochondria of tumour cells rather than those of normal cells, and thus exhibit lower toxicity. In addition, thiosemicarbazone complexes achieve their therapeutic effects via a different mechanism of action than traditional chemotherapeutic drugs; thus, these complexes may overcome the problem of multidrug resistance. Many *in vitro* cell experiments and animal model studies have demonstrated the efficacy and safety of thiosemicarbazone metal complexes, which exhibit inhibitory effects on various tumour types and will therefore inform future clinical studies. Specific thiosemicarbazone metal complexes can penetrate the cell membrane and accumulate in the inner mitochondrial membrane, whereupon they reduce the mitochondrial membrane potential and trigger apoptosis. The catalytic activity of certain thiosemicarbazone metal complexes generates a plethora of reactive oxygen species, which, in excess, can impair mitochondrial DNA and proteins, thereby accelerating cellular apoptosis. By disrupting the activity of crucial enzymes in the electron transport chain, these complexes impede the mitochondrial respiratory chain function and hinder ATP synthesis, ultimately depleting the energy reservoir of tumour cells. Thiosemicarbazone metal complexes can also regulate the permeability of the mitochondrial outer membrane, release cytochrome C and other apoptotic factors, regulate Bcl-2 family proteins, initiate the apoptotic cascade reaction, and cause apoptosis.

Despite the advantages of these complexes, challenges associated with improving the stability and bioavailability, optimising drug delivery strategies, and gaining a deep understanding of their mechanisms of action and potential side effects currently impede their widespread clinical use. Future research will optimise the design of these complexes to improve their selectivity, bioavailability, and stability. Additional studies exploring their mechanisms of action, along with preclinical and clinical trials, will transform these compounds into clinically usable antitumour agents. Given the abnormal dependence of tumour cells on glycolysis, drugs have been developed to disrupt tumour-specific metabolic pathways, such as the tricarboxylic acid cycle or oxidative phosphorylation processes. The programmed death of tumour cells is directly triggered by compounds that can open the mitochondrial outer membrane permeability conversion pore and release cytochrome C and other apoptosis-inducing factors. Further development of metal complexes and small-molecule drugs is required to improve their ability to increase ROS levels in tumour cells, which leads to excessive oxidative stress and subsequently induces apoptosis or autophagy.

The use of thiosemicarbazone metal complexes as antitumor agents that target mitochondria is an innovative strategy in the fight against cancer. Further in-depth investigations into the mechanism of action and clinical utility of such complexes is expected to bring revolutionary progress in tumour therapy.
